# Cardiac stem cells in patients with heart disease

**DOI:** 10.3892/etm.2013.984

**Published:** 2013-02-28

**Authors:** XIAOHUI ZHAO, LAN HUANG

**Affiliations:** Department of Cardiology, Xinqiao Hospital, Third Military Medical University, Chongqing 400037, P.R. China

**Keywords:** cardiac stem cells, myocardial regeneration, heart disease

## Abstract

The heart has been regarded as a terminally differentiated organ for decades. There are numerous indicators for the potency of myocardial regeneration, which opens up new avenues for the treatment of heart disease. Cardiac stem cells (CSCs) have been discovered in the human heart and they play a vital role in myocardial regeneration. This review discusses the distribution, properties and proliferation of CSCs in the myocardium of patients with heart disease. Additionally, the potency of myocardial regeneration in patients with heart disease is discussed.

## Contents

1. Introduction
2. Regeneration processes in human myocardium
3. Characteristics of CSCs
4. Properties and proliferation of CSCs in the myocardium of patients with heart disease
5. Potency of myocardial regeneration in patients with heart disease
6. Origin of the c-kit^+^ CD34^−^CD45^−^ cells and Ki-67^+^ cardiomyocytes in the myocardium of patients with heart disease
7. Origin of Ki-67^+^ cardiomyocytes and c-kit^+^ CD34^−^CD45^−^ cells in patients with aortic stenosis
8. Conclusions

## 1. Introduction

Cell therapy in cardiology is the process of transplantation of sufficient cells into a particular myocardial area, ensuring the survival and integration of the transplanted cells. Thus, cells for cell therapy should be multipotent and non-immunogenic, without oncogenic potency. Several types of stem cells have been discovered, including embryonic stem cells (ESCs), hematopoietic stem cells, mesenchymal stem cells (MSCs), bone marrow stem cells (BMSCs) and cardiac stem cells (CSCs). However, a suitable stem cell type for cell therapy in cardiology has not yet been identified.

Previously, it was assumed that the loss of cardiomyocytes was irreversible, a hypothesis based on the generally accepted doctrine that cardiomyocytes are subject to terminal differentiation after birth and no longer participate in the cell cycle (1). However, myocardial regeneration in the human heart and the discovery of CSCs have led to a new paradigm in cell therapy.

Thus, this review discusses the distribution, properties and proliferation of CSCs in the myocardium and the potency of myocardial regeneration in patients with heart disease. In addition, the regeneration processes in the human myocardium are also discussed.

## 2. Regeneration processes in human myocardium

**Figure 1:**
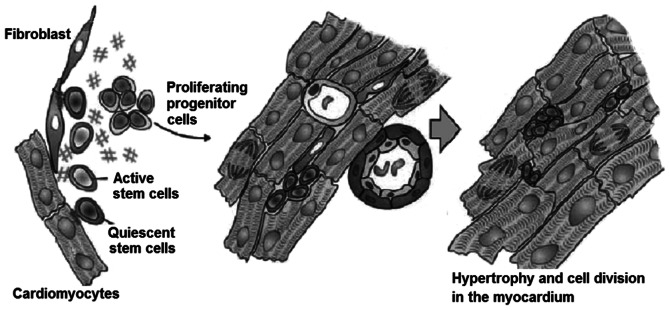
Cardiac stem cell niches in the myocardium. Following activation, these cells develop into cardiomyocytes and vascular structures.

Previously, it was assumed that the loss of cardiomyocytes is irreversible and that hypertrophy of remaining cardiomyocytes is the only compensation for loss of cells in the heart. These assumptions were based on the generally accepted doctrine that cardiomyocytes were subject to terminal differentiation after birth and no longer participated in the cell cycle (1). However, evidence of myocardial regeneration in the human heart has led to a new paradigm. Consequently, the heart may not be a terminally differentiated organ and may have a higher regenerative capacity than previously assumed (Fig. 1) (2).

One study demonstrated that cardiomyocytes possess regenerative potential in the adult heart, as well as in animal hearts (3). Anversa *et al* first provided evidence of regenerative processes in the human heart following cardiac transplantation (4). The transplanted heart clearly contained immigrant cardiomyocyte cells with surface markers. The origin of these newly-formed cells are the progenitor cells of the primary heart, in addition to circulating progenitor cells from the untreated atrial remnants of the receiver (5). Further research revealed the existence of CSCs in the myocardium of rats, dogs and humans. In particular, the authors identified a cell population, which were the marker molecules of adult stem cells. However, these markers were not expressed by hematopoietic cells. The cell population was described as CSCs (4).

## 3. Characteristics of CSCs

**Figure 2:**
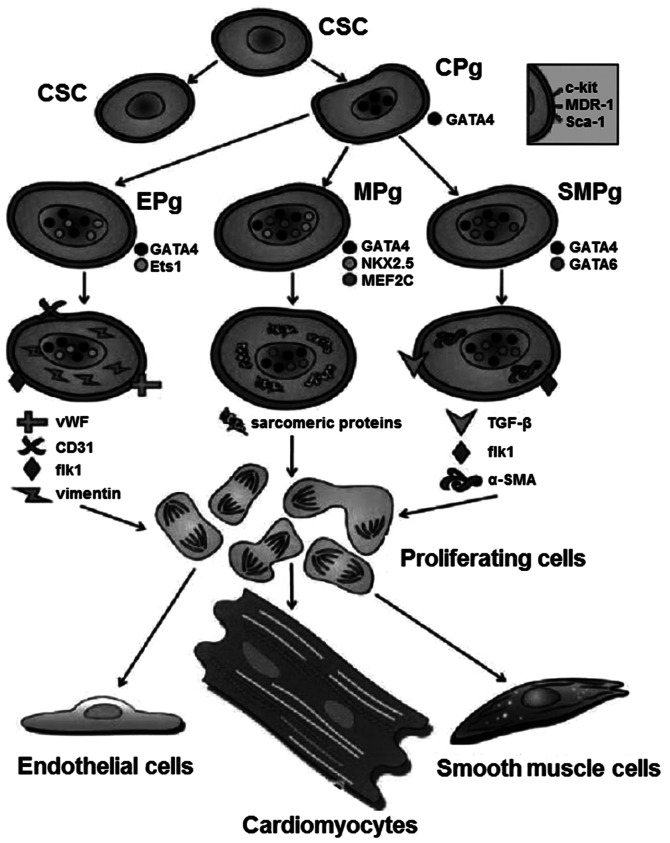
Growth and differentiation of cardiac stem cells (CSCs).

Rodent models of myocardial infarction have revealed that CSCs have a potential for proliferation and myocardial regeneration (7). In animal studies, these cells are differentiated into cardiac cell types, including cardiomyocytes, endothelial cells and smooth muscle cells (Fig. 2) (4). This basic hypothesis was strengthened by the results of other research groups, that described other cell populations with the characteristics of adult CSCs and the methods for the isolation and expansion of CSCs (5,7–13). Therefore, it is important to meet the prerequisites for the application of this cell population in cell therapy. The surface markers of CSCs, including stem cell growth factor receptor (c-kit), a transporter protein (multidrug resistance protein 1; MDR-1) and a stem cell antigen (Sca-1) have been detected (4). Furthermore, the expression of cardiac transcription factors, including the cardiac myosin and cytoplasmic proteins have also been detected in CSCs. Hematopoietic or bone marrow cell markers were not identified in CSCs (12). Further research is required to clarify whether similar cell populations also exist in human tissues and whether they play a role in pathology. Due to their local resistance, CSCs are the optimal choice for cell replacement therapy. Among all the cell populations, they are the most similar to cardiomyocytes and easily integrate into the myocardium. One study demonstrated that CSCs are able to differentiate into cardiomyocytes and vascular cells, which is a prerequisite for a viable myocardium. Furthermore, CSCs are easily isolated and duplicated in cell culture (5). This fact predisposes the cell population of CSCs for cell therapy. A limited factor may be the extremely small number of CSCs available. To date, it remains unclear how CSCs may be multiplied to produce sufficient numbers.

## 4. Properties and proliferation of CSCs in the myocardium of patients with heart disease

Studies of the myocardium of various animal species, including mice (9), rats (6), dogs (14) and pigs (15), revealed the presence of a c-kit^+^ CD34^−^CD45^−^ cell population, which are similar to CSCs in the human myocardium of patients with heart disease (4).

c-kit is the receptor of stem cell growth factor and a marker for the detection of CSCs, since c-kit^+^ plays an important role in regeneration processes of the human heart and has the ability to regenerate myocardial cells (16,17). Anversa *et al* demonstrated that c-kit^+^ cardiac cells are clonogenic multipotent cells with the ability to self-renew and differentiate into at least three different cardiogenic cell lines, including myocytes, smooth muscle cells and endothelial cells (18). An animal model demonstrated that c-kit^+^ cardiac cells are able to regenerate functional myocardium *in vivo*, which was not produced through cell fusion (5). These cells also succeeded in the differentiation of three different cardiogenic cells in *in vitro* beating myocytes (9). Thus, c-kit^+^ cardiac cells demonstrated the potential for regeneration of the various components of the myocardium (5,10,12). In addition to c-kit, Anversa *et al* identified the population of CSCs by the two surface markers MDR-1 and Sca-1 (5). Urbanek *et al* also examined three markers, c-kit, Sca-1 and MDR-1, on CSCs. The authors demonstrated that ∼60% of CSCs expressed all three markers and 80% of CSCs were c-kit^+^(19). Thus, the population of CSCs that are c-kit^+^ is large. The absolute numbers of the total CSC population may be identified using c-kit as a marker. The potential and exact properties of these cells were investigated further *in vivo* and *in vitro*(20,21).

It is known that c-kit, as a marker of hematopoietic and endothelial stem cells and mast cells, may also be expressed in the myocardium. Other studies identified that heart cells with surface markers of stem cells did not express the hematopoietic or endothelial markers (12,13,22).

The number of c-kit^+^ CD34^−^CD45^−^ cells has been determined. The number of c-kit^+^ CD34^−^CD45^−^ cells in patients with dilated cardiomyopathy, heart failure and aortic stenosis was 0.19–0.21 cells/mm^2^(13,23). The average number of c-kit^+^ MDR-1-Isl-1 CD45^+^ cells was 2.7±1.3 in human myocardium biopsies (17). Beltrami *et al* reported one stem cell per 10,000 cardiomyocytes in rats (5) and CSCs were 0.56 per 10,000 cardiomyocytes in the myocardium of dogs (14). Further studies identified a range of CSCs per 8,000–80,000 cardiomyocytes in mice, rats, dogs and humans (4). Although all the studies used c-kit as a stem cell marker, a number of studies used MDR-1 or Sca-1 (5,16). Moreover, not all CD34^+^ and CD45^+^ cells were excluded (5,17). However, CSCs are relatively rare. It is generally assumed that adult stem cells are rare, regardless of tissue (24). Thus, hematopoietic c-kit^+^ CD34^+^ stem cells, which were discovered ∼40 years ago and therefore extremely well studied, were estimated at 1:10,000–1:15,000 (24).

## 5. Potency of myocardial regeneration in patients with heart disease

Studies have identified that myocardial regeneration occurs not only in acute pathological conditions, including following acute myocardial infarction, but also in chronically damaged hearts (13,19). It was observed that patients with heart failure or idiopathic dilated cardiomyopathy had significantly more c-kit^+^ CD34^−^CD45^−^ cells than control subjects without cardiac disease (23). Several studies demonstrated the activation and proliferation of CSCs in damaged heart tissue (5,13,19,23). The occurrence of regenerative processes in pathologically altered myocardium suggests an increased number of CSCs, as well as an increased proliferative activity in the myocardium (7,10). It is interesting that the number of other CSC populations, including the number of Sca-1^+^ cells in the myocardium increases when the adult heart is subjected to a load (25). The increased expression of Sca-1 in cells of the hypertrophic myocardium leads to an increased demand for Sca-1^+^ cells (25).

Studies have revealed cardiac regeneration processes in pathologically altered myocardium; however, the regeneration process is not clinically apparent. The small number of c-kit^+^ CD34^−^CD45^−^ CSCs, with an average of 1.3±1.3 CSCs per 40,000 cardiomyocytes, may not be sufficient; therefore, the functionally effective regeneration processes to compensate for serious damage in the myocardium remain uncertain.

Studies have shown that the number of CSCs in patients with chronic ischemic heart damage is lower than those with acute myocardial infarction (19). The formation of new cardiomyocytes is weakened by prolonged and end-stage heart failure decompensation (3,26). However, an increased number of Ki-67^+^ cardiomyocytes was observed in terminal heart failure patients who received a heart transplant. Therefore, regeneration processes may be secondary to myocardial apoptosis. Urbanek *et al* observed an increase in apoptotic processes of CSCs from 0.3 in healthy to 9.6% in chronically damaged myocardium. The authors identified that heart failure patients have a higher number of p53-positive senescent CSCs with short telomeres. p53 and telomere shortening are markers of cellular aging processes (27). p53 induces growth arrest and cellular senescence via cyclin-dependent kinase (CDK)4 and CDK6, which block the retinoblastoma protein in its active hypophosphorylated state (28). This loss of functionally competent CSCs in chronic ischemic damage is the progressive loss of function in terminal heart failure (19). It is of note that c-kit^+^ CD34^−^CD45^−^ cells could be detected in the myocardium of heart failure patients and healthy individuals (23). An increase of c-kit^+^ CD34^−^CD45^−^ cells was observed in the damaged heart. However, 59% of the c-kit^+^ CD34^−^CD45^−^positive cells cells were p16^INK4a^-positive in the damaged heart, but only 14% of the c-kit^+^ CD34^−^CD45^−^ cells expressed p16^INK4a^ in the normal myocardium of control patients (23). The c-kit^+^ cells cells significantly increased in the myocardium of patients with heart failure (23).

An interesting aspect is the consequence of a sudden interruption of blood supply in all organs, regardless of whether an institution possesses proliferation capacity. The kidneys are known to have cells that are able to re-enter the cell cycle and actively proliferate (29). However, a heart attack results in cell death, tissue loss and scar formation in the ischemic region. Thus, the self-renewal potential of the heart is due to cell regeneration from stem cells. Slowly progressive lesions are compensated by this pool of cells before they enter into clinical appearance (13,30).

## 6. Origin of the c-kit^+^ CD34^−^CD45^−^ cells and Ki-67^+^ cardiomyocytes in the myocardium of patients with heart disease

A significantly increased number of c-kit^+^ CD34^−^CD45^−^ cells was observed in the myocardium of 19 heart failure patients compared with seven healthy control subjects (23). A ten-fold increase in the number of dividing cardiomyocytes in heart failure was observed in another study (19). If we define the dividing cardiomyocytes by the presence of the marker Ki-67, there is an increase of at least four times in patients with heart failure.

Based on the increased detection of c-kit^+^ CD34^−^CD45^−^CSCs and Ki-67^+^ cardiomyocytes with terminal heart failure, the question arises as to the origin of these cells. The fundamental problem regarding adult stem cells is the lack of definition. Unlike embryonic stem cells, which are defined by their origin from the inner cell mass of the blastocyst, there is no exact definition for adult stem cells. The real origin of adult stem cells, independent of tissue is unknown (5). Thus, the origin of the increased number of c-kit^+^ CD34^−^CD45^−^ cardiac cells and Ki-67^+^ cardiomyocytes with terminal heart failure was interpreted in several ways: i) activation of CSCs, which increases the total number and proliferative activity of the myocardium (5,13,14,19); ii) immigration of non-CSCs; iii) lack of differentiation of progenitor cells under pathological conditions and thus accumulation of juvenile cardiomyocytes; iv) dedifferentiation and expression of fetal and embryonic molecules of damaged adult cardiomyocytes.

In relation to ii), the detection of CD34^−^CD45^−^cells in the myocardium of heart failure patients may involve immigrant endothelial or hematopoietic progenitor cells lagging behind their migration into the myocardium using their endothelial and hematopoietic markers (31).

## 7. Origin of Ki-67^+^ cardiomyocytes and c-kit^+^ CD34^−^CD45^−^cells in patients with aortic stenosis

To analyze the cellular levels in the left ventricle of the heart, a large number of samples should be collected to obtain statistically reliable results. Approximately half the total number of c-kit^+^ CD34^−^CD45^−^ or Ki-67^+^ cardiomyocytes are present in the left ventricle of patients with aortic stenosis. The myocardium of the left ventricle is involved in the pathogenesis of this disease and if these cells were identified here, the regeneration processes would be expected, particularly since the rate of newly formed myocytes increases with an increase in wall stress (7,10). The reason for this discrepancy may be in the evaluation of a given area that is exposed, particularly in aortic stenosis of the left ventricle, to chronic pressure load, resulting in the development of concentric hypertrophy (30). Consequently, larger and fewer cells may have been included in the analysis. Nevertheless, more c-kit^+^ Lin^−^ and Ki-67^+^ cells were detected in samples from the outflow tract of 36 patients with relevant aortic valve stenosis and valve replacement compared with 12 control subjects who succumbed to a non-cardiac cause (13). An increase in Ki-67^+^ cardiomyocytes was also reported compared with c-kit^+^ CD34^−^CD45^−^ cells. The growth and differentiation of stem cells into mature cardiac myocytes in the hypertrophied myocardium of patients with chronic aortic valve was associated with the fact that patients with aortic stenosis have good ventricular function for a number of years before decompensation becomes visible (32). It is known that patients with aortic stenosis typically remain asymptomatic for a long period (30).

These patients also had a good pump function [left ventricular ejection fraction (LVEF), 53.2±13.2%]. This suggests that small, gradual restriction of ventricular function may be balanced in contrast to fulminant ischemia. This regeneration process may contribute to hemodynamic changes and remain stable over long periods.

## 8. Conclusions

The heart has long been regarded as a terminally differentiated organ. However, there are numerous indicators of potential regeneration of the myocardium. Previously, CSCs were discovered in the human heart and have been attributed to a particular role. Cell therapy in cardiology is a new method for the treatment of patients with heart disease. However, a suitable stem cell type has not yet been identified for cell therapy in cardiology. This review discusses the distribution, properties and proliferation of CSCs in the myocardium. The potency of myocardial regeneration and the regeneration processes in patients with heart disease were also discussed.
